# 胸外科住院肺癌合并慢性阻塞性肺疾病的调查结果分析

**DOI:** 10.3779/j.issn.1009-3419.2017.03.04

**Published:** 2017-03-20

**Authors:** 荣葆 张, 星宇 谭, 清 陈, 敬安 魏, 军 改, 妍 王, 子逸 杨, 晶 李, 丽宇 祝, 仲贤 黄, 权瀛 何

**Affiliations:** 1 100044 北京，北京大学人民医院呼吸与危重症科 Department of Respiratory and Critical Care, Beijing 100044, China; 2 100044 北京，教育处8年制医学生 Education Division, People's Hospital Peking University, Beijing 100044, China

**Keywords:** 性阻塞性肺疾病, 筛查, 肺肿瘤, 肺功能试验, Chronic obstructive pulmonary disease, Screening, Lung neoplasms, Lung function tests

## Abstract

**背景与目的:**

肺癌是慢性阻塞性肺疾病（慢阻肺）重要的合并症，会显著影响慢阻肺患者的预后。慢阻肺也会影响肺癌患者的术后并发症和复发。本研究旨在调查胸外科住院肺癌合并慢阻肺的情况。

**方法:**

回顾性分析北京大学人民医院胸外科2015年1月-2015年12月收治的原发性非小细胞肺癌患者。通过查阅病历获取患者的性别、年龄、吸烟状况、有害职业史、症状、胸部计算机断层扫描（computed tomography, CT）、术后病理、出院诊断、全套肺功能及支气管舒张试验，当基础肺功能第一秒钟用力呼气容积（forced expiratory volume in one second, FEV_1_）占预计值＜70%时即行支气管舒张试验。

**结果:**

703例肺癌患者进行了全套肺功能测定，其中67例进行支气管舒张试验，62例（92.5%）符合慢阻肺诊断。接受手术治疗的肺癌患者有677例，其中41例进行支气管舒张试验，38例（92.7%）符合慢阻肺诊断。在接受手术治疗的肺癌患者中合并慢阻肺者年龄≥65岁、男性、有吸烟史和非腺癌的比例高于未合并慢阻肺者，差异有统计学意义（*P*＜0.05）；男性和≥65岁者更易合并慢阻肺（OR: 2.807-2.374, 95%CI: 1.101-7.157）（*P*＜0.05）。住院前仅有3例（4.3‰）诊断慢阻肺并按慢阻肺规范治疗。出院时仅有5例（7.1‰）诊断慢阻肺。

**结论:**

在胸外科住院肺癌患者中行常规肺功能及支气管舒张试验可提高肺癌合并慢阻肺的诊断；当前肺癌合并慢阻肺诊断和治疗严重不足，需要引起胸外科医生重视，与呼吸内科医生携手共同防治慢阻肺。

## Abstract (en)

**Background and objective:**

Lung cancer is an important complication of chronic obstructive pulmonary disease (COPD), and even significantly affects the prognosis of patients with COPD. COPD also affects the postoperative complications and recurrence in patients with lung cancer. This study aims to investigate lung cancer patients complicated with COPD in thoracic surgical department.

**Methods:**

All medical records of lung cancer patients discharged from the Department of Thoracic Surgery of People's Hospital, Peking University during January 2015 and December 2015 were reviewed, including gender, age, tobacco smoke history, harmful occupational exposure, clinic symptom, chest computed tomography (CT) scanning, postoperative pathology result report, discharged diagnosis and spirometry [All patients underwent pulmonary function test are received bronchial dilation test if the based predicted value of forced expiratory volume in one second (FEV_1_) < 70%].

**Results:**

A full set of lung function test was measured in 703 lung cancer patients. Bronchial dilation test was finished in 67 patients. 62 (92.5%) patients were diagnosed as COPD. 677 cases with lung cancer were received surgery. Bronchial dilation test was measured in 42 cases. Of them 38 (92.7%) patients were diagnosed as COPD. It was found that the patients with lung cancer and COPD was more frequent in males, elders (≥65 yr), smokers, non-adenocarcinoma patients than those of patients without COPD (*P* < 0.05). The males and the elders (≥65 yr) were more likely to suffer from COPD (OR: 2.374-2.807, 95%CI: 1.101-7.157)(*P* < 0.05). Only 3 patients (4.3‰) were diagnosed as COPD and received standard treatment before admission. And only 5 patients (7.1‰) were diagnosed as COPD as discharged.

**Conclusion:**

The routine pulmonary function as well as bronchial dilation test are helpful for screening the patients with COPD. At present, the diagnosis and treatment of lung cancer combined with COPD is a serious problem, which needs to be paid attention to by thoracic surgeons and to join hands with physicians in order to improve the diagnosis level of COPD.

慢性阻塞性肺疾病（慢阻肺）是一种常见的慢性呼吸道疾病。我国40岁以上人群中慢阻肺患病率为8.2%，我国现有慢阻肺人数约为3, 800万-4, 200万^[1]^。大量的证据^[2-4]^表明慢阻肺与肺癌密切相关，二者常常并存或先后发生。长期大量吸烟和高龄既是慢阻肺的主要危险因素，同时又是肺癌，特别是肺鳞癌的主要高危因素。2011年《慢性阻塞性肺疾病全球倡议》（global initiative for chronic obstructive pulmonary disease, GOLD）已将肺癌列为慢阻肺重要合并症，肺癌会显著影响慢阻肺的病情严重度，并且认为是引起慢阻肺死亡的重要原因之一^[5]^。肺癌患者中有相当大一部分人需要接受手术治疗，为了准确掌握外科手术适应证，评估术中风险以及术后患者的预后、恢复情况，绝大多数患者均要进行肺功能测定，包括肺容积、通气功能、换气功能测定，重点是肺储备功能。这就给我们提供了从肺癌患者中主动发现慢阻肺的良机。只要在肺功能测定项目中加入支气管舒张试验，再结合病史、体检和影像学资料即可明确肺癌合并慢阻肺的诊断。为了验证这一设想，2015年1月1日-12月31日我们对所有胸外科住院行肺功能测定的患者，当其基础第一秒钟用力呼气容积（forced expiratory volume in one second, FEV_1_）占预计值＜70%时即行支气管舒张试验以调查慢阻肺，现将结果报告如下。

## 1 资料与方法

### 1.1 纳入和排除标准

纳入标准：①2015年1月-2015年12月北京大学人民医院胸外科收治的非小细胞肺癌（non-small cell lung cancer, NSCLC）患者；②所有患者均经组织病理学确诊为NSCLC，并按美国癌症联合委员会（American Joint Committee on Cancer, AJCC）第7版判定分期标准。排除标准：痰液细胞学诊断的NSCLC者。

### 1.2 慢阻肺诊断标准

根据2015年《慢性阻塞性肺疾病防治全球创议》（The Global Initiative for Chronic Obstructive Lung Disease, GOLD）^[5]^和2013年中国慢阻肺诊治指南^[6]^，即任何有呼吸困难、慢性咳嗽或咳痰，且具有吸烟史和（或）环境职业污染及生物燃料接触史，吸入支气管舒张剂后FEV_1_/用力肺活量（forced vital capacity, FVC）＜70%即明确存在持续气流受限，除外其他疾病后可确诊为慢阻肺。本调查根据病案记录，综合患者的吸烟史、职业史、临床表现、基础肺功能和支气管舒张试验以及胸部计算机断层扫描（computed tomography, CT），排除支气管扩张、支气管哮喘、充血性心力衰竭、闭塞性支气管炎、肺结核、弥漫性泛细支气管炎等引起气流受限的疾病，临床诊断为慢阻肺。

### 1.3 气流受限严重程度的肺功能分级^[6]^

根据吸入支气管舒张剂后FEV_1_占预计值的百分比（FEV_1_%预计值）将气流受限程度分为4级：Ⅰ级（FEV_1_%预计值≥80%）、Ⅱ级（50%≤FEV_1_%预计值＜80%）、Ⅲ级（30%≤FEV_1_%预计值＜50%）、Ⅳ级（FEV_l_%预计值＜30%）。

### 1.4 支气管舒张试验方法

根据2014年中华医学会呼吸病学分会肺功能专业组制定肺功能检查指南（第四部分）——支气管舒张试验^[7]^，先行基础肺功能检查并确定符合质量标准，如果FEV_1_占预计值%＜70%即行支气管舒张试验。吸入定量气雾剂沙丁胺醇400 μg，在吸入药物后15 min，再复查用药后肺功能。FEV_1_用药后较用药前增加≥12%，且绝对值增加≥200 mL，则为支气管舒张试验阳性。

### 1.5 肺癌诊断标准

根据美国国立综合癌症网络（National Comprehensive Cancer Network, NCCN）肿瘤学临床实践指南的2014年NSCLC诊疗指南^[8]^。

### 1.6 临床资料的收集

通过查阅病历获取患者的临床基线特征及检查治疗信息。本研究收集以下数据：性别、年龄、吸烟状况、有害职业史、症状、胸部CT、术后病理、出院诊断、全套肺功能及支气管舒张试验。所有患者当基础肺功能FEV_1_占预计值＜70%时即行支气管舒张试验。

### 1.7 统计学方法

用Epidata 3.0软件进行数据录入，双人录入并进行一致性检验后，导入SPSS 19.0（SPSS Inc., Chicago, IL, USA）软件进行数据统计分析。其中计量资料采用Mean±SD表示，计数资料用例数（构成比）表示。利用*t*和*χ*^2^检验比较手术治疗肺癌合并/未合并慢阻肺患者的临床特征差异，将性别、年龄、吸烟纳入多因素*Logistic*回归，分析肺癌合并慢阻肺的影响因素。*P*＜0.05为差异具有统计学意义。

## 2 结果

### 2.1 胸外科住院肺癌患者合并慢阻肺的情况

全年共有703例肺癌患者进行了全套肺功能测定，其中19例患者吸入支气管舒张剂前FEV_1_/FVC＜70%，因其FEV_1_占预计值%≥70%，未做支气管舒张试验。进一步分析这些患者的肺功能测定结果和临床资料，其中有4例残气和/或肺总量占预计值%＞120%；有5例弥散功能（diffusing capacity of the lung for carbon monoxide, DLCO）占预计值%＜80%；两者均异常的有4例。有17例胸部高分辨率CT显示有不同程度肺气肿征象。67例行支气管舒张试验的患者中有62例（92.5%）符合慢阻肺诊断，按气流受限严重程度的肺功能分级，Ⅰ级5例，Ⅱ级46例，Ⅲ级8例，Ⅳ级3例。合并慢阻肺的肺癌中鳞癌38例，腺癌22例，大细胞癌1例，支气管粘液表皮样癌1例。

### 2.2 胸外科住院手术治疗的肺癌患者合并慢阻肺的情况

手术治疗的肺癌患者有677例，其中41例行支气管舒张试验，38例（92.7%）符合慢阻肺诊断，按气流受限严重程度的肺功能分级Ⅰ级3例，Ⅱ级28例，Ⅲ级5例，Ⅳ级2例。

### 2.3 肺癌合并/未合并慢阻肺的临床特征

合并慢阻肺组年龄[（65.8±7.72）岁]高于未合并慢阻肺组[（60.0±11.00）岁]（*P*＜0.05）。合并慢阻肺组年龄≥65岁23例（60.5%）、男性30例（78.9%）、有吸烟史21例（55.3%），非腺癌13例（41.9%）均高于未合并慢阻肺组，差异有统计学意义（均*P*＜0.05），合并慢阻肺组分期为Ⅱ期及以下（0期或Ⅰ期或Ⅱ期）20例（64.5%）、Ⅲ期及以上（Ⅲ期或Ⅳ期）11例（35.5%）与未合并慢阻肺组比较无统计学差异（表 1）。

**1:** 合并与未合并慢阻肺手术治疗肺癌患者的临床特征比较 Clinical characteristics of surgical treated lung cancer patients (with/without COPD)

Characteristics	With COPD [*n* (%)]	Without COPD [*n* (%)]	*χ*^2^	*P*	OR	95%CI
Age (yr) (*n*=677)						
≥65	23 (60.5)	235 (36.8)	8.578	0.003	1.646	1.249-2.169
＜65	15 (39.5)	404 (63.2)			1.000	
Gender (*n*=677)						
Male	30 (78.9)	329 (51.5)	10.859	0.001	1.533	1.280-1.837
Female	8 (21.1)	310 (48.5)			1.000	
Smoking history (*n*=649)						
Yes	21 (55.3)	209 (34.2)	6.933	0.008	1.616	1.189-2.195
No	17 (44.7)	402 (65.8)			1.000	
Tumor pathological type (*n*=562)						
Non-adenocarcinoma	13 (41.9)	112 (21.1)	7.357	0.007	1.988	1.273-3.105
Adenocarcinoma	18 (58.0)	419 (78.9)			1.000	
Pathological stage (*n*=582)						
0 or Ⅰ orⅡ	20 (64.5)	393 (71.3)	0.660	0.416	0.905	0.693-1.181
Ⅲ or Ⅳ	11 (35.5)	158 (28.7)			1.000	
COPD: chronic obstructive pulmonary disease.						

### 2.4 将年龄、性别和吸烟史纳入多因素*Logistic*回归模型进行多因素分析

各变量赋值如下：性别：男=1，女=2；年龄：＜65岁=1，≥65岁=2；吸烟史：吸烟=1，不吸烟= 2。多因素分析显示，与女性相比，男性更易合并慢阻肺（OR=2.807, 95%CI: 1.101-7.157）；与＜65岁的相比，≥65岁者更可能合并慢阻肺（OR=2.374, 95%CI: 1.204-4.68）（均*P*＜0.05）（表 2）。

**2:** 多因素*Logistic*分析影响肺癌患者合并慢阻肺的独立因素 Independent factors of lung cancer with COPD by multivariate *Logistic* analysis

Characteristic	*β* (B)	SE	Wald	*P*	OR	RR (95%CI)
Gender						
Male	-1.032	0.477	4.674	0.031	2.807	1.101-7.157
Fmale					1.000	
Age (yr)						
≥65	0.865	0.346	6.233	0.013	2.374	1.204-4.681
＜65					1.000	
Smoking history						
Yes	-0.234	0.398	0.346	0.556	1.264	0.579-2.760
No					1.000	

### 2.5 慢阻肺的诊断情况

住院前仅3例（4.3‰）诊断慢阻肺，并按慢阻肺规范治疗。出院时仅5例（7.1‰）诊断慢阻肺。

## 3 讨论

肺癌合并慢阻肺的调查十分必要。有研究^[9]^表明术后肺癌复发率与慢阻肺严重度密切相关。肺癌术后未复发5年生存率在无慢阻肺、轻度慢阻肺和重度慢阻肺中分别为78.1%、70.4%和46.4%（*P*=0.001）。因此，胸外科医生应关注慢阻肺，关注慢阻肺与肺癌的相关性。

有研究报告^[10]^显示50%-70%的肺癌患者合并慢阻肺，特别是鳞癌患者中合并慢阻肺的比率更高，其主要机制在于慢阻肺与肺癌具有很多相同、相似的病因或高危因素，例如长期吸烟、空气污染、从事某些有害职业（如煤尘、矽尘暴露史）、全身炎症等，提示我们可以从肺癌患者中主动发现慢阻肺。2015年1月-12月我们对所有行肺功能测定的胸外科住院患者，当其基础FEV_1_占预计值%＜70%时即行支气管舒张试验，从703例肺癌患者中检出了62例（8.8%）慢阻肺。可见从肺癌患者中筛查慢阻肺是可行的。而且花费不大，每一次支气管舒张试验检查费仅45元，的确是一种简便、价廉的好办法。

本研究显示在手术治疗的肺癌患者中，年龄≥65岁、男性、有吸烟史，非腺癌者合并慢阻肺的比例高于未合并者。这与其他学者^[9]^的研究是一致的。多因素分析显示，≥65岁和男性更易合并慢阻肺；因此，应着重在老年、男性、吸烟和非腺癌患者中开展慢阻肺的筛查。

胸外科医生平时临床上的着眼点和注意力主要放在术前诊断、手术指证的掌握、术式选择、手术安全性、术后病理、术后伤口愈合及康复等，当然也会关注术后合并症。但是对于患者并存的其他疾病关注度不像主要疾病那么强烈。复旦大学中山医院的调查^[11]^显示呼吸科医生对于慢阻肺的诊断率（34.8%）明显高于非呼吸科医生（2.9%）。国外学者的研究^[12]^表明只有约10%合并慢阻肺的肺癌患者接受了慢阻肺规范治疗。本研究显示尽管有62例患者肺功能符合慢阻肺的诊断，然而住院前仅3例（4.3‰）诊断慢阻肺，并按慢阻肺规范治疗，出院时仅5例（7.1‰）诊断慢阻肺。由此可见，胸外科住院的肺癌合并慢阻肺诊断与治疗关注不足。对于慢阻肺对肺癌患者的康复、预后的影响缺乏应有的重视，胸外科医生应重视肺癌合并慢阻肺问题。建议肺癌患者入院后除行常规肺功能测定还应提交支气管舒张试验的申请当结果回报后请呼吸科医师会诊，明确诊断，并制定规范的治疗方案，出院后在胸外科和呼吸科随访。

由于本研究是回顾性分析，资料来源于胸外科出院病历，使得纳入的肺癌合并慢阻肺的数量和影响因素受到了一定限制，仅对患者的性别、年龄、吸烟史、肺癌病理类型和分期进行了分析。

本文报告的患者都是在完成常规肺功能测定后，当FEV_1_占预计值%＜70%时才行支气管舒张试验。其中有19例患者吸入支气管舒张试验前FEV_1_占预计值%≥70%，但FEV_1_/FVC＜70%，其后又未做支气管舒张试验。这些患者中有可能存在慢阻肺，但是由于我们没有进行支气管舒张试验，因而无法进一步作出判断。我们曾对393例门诊因症就医患者进行了肺功能和支气管舒张试验，发现舒张前FEV_1_≥70%预计值而FEV_1_/FVC＜70%有49例，占393例的12.5%，舒张试验后FEV_1_/FVC仍＜70%的有33例，占393例的8.4%（论文待发表）。由此我们推断，照目前的方法，这19例患者中可能有些慢阻肺被漏诊。

## References

[1] Zhong N, Wang C, Yao W (2007). Prevalence of chronic obstructivepulmonary disease in China: a large, population-based survey. Am J Respir Crit Care Med.

[2] Lange P, Nyboe J, Appleyard M (1990). Ventilatory function and chronic mucus hypersecretion as predictors of death from lung cancer. Am Rev Respir Dis.

[3] Tockman MS, Anthonisen NR, Wright EC (1987). Airways obstruction and the risk for lung cancer. Ann Intern Med.

[4] de-Torres JP, Wilson DO, Sanchez-Salcedo P (2015). Lung cancer in patients with chronic obstructive pulmonary disease. Development and validation of the COPD Lung Cancer Screening Score. Am J Respir Crit Care Med.

[b5] 5 Global strategy for the diagnosis, management and preventionofchronic obstructive pulmonary disease (updated 2015)[EB/OL]. [2015-01]. <a href="http://www.goldcopd.org/guidelines-globalstrategy-for-diagnosis-management.html" target="_blank">http://www.goldcopd.org/guidelines-globalstrategy-for-diagnosis-management.html</a>.

[6] Chronic Obstructive Pulmonary Disease Committee, Respiratory Society, Chinese Medical Association (2013). The guideline for diagnosis and management of chronic obstructive pulmonary disease (revised in 2013). Zhonghua Jie He He Hu Xi Za Zhi.

[7] Lung Function Committee, Respiratory Society, Chinese Medical Association (2014). Guidelines for pulmonary function tests (Part Ⅳ)-bronchial dilation test. Zhonghua Jie He He Hu Xi Za Zhi.

[8] Liu XQ, Guo WF, Chen KN (2014). Research progress increases the pace of clinical practice revolution, interpretation of updates of 2014 NSCLC guideline. Zhejiang Yi Xue.

[9] Qiang GL, Liang CY, Xiao F (2016). Impact of chronic obstructive pulmonary disease on postoperative recurrence in patients with resected non-small-cell lung cancer. Int J Chron Obstruct Pulmon Dis.

[10] Young RP, Hopkins RJ, Christmas T (2009). COPD prevalence is increased in lung cancer independent of age, sex and smoking history. Eur Respir J.

[11] Zhang J, Zhou JB, Lin XF (2013). Prevalence of undiagnosed and undertreated chronic obstructive pulmonary disease in lung cancer population. Respirology.

[12] Gottlieb M, Marsaa K, Godtfredsen NS (2015). Prevalence and management of pulmonary comorbidity in patients with lung and head and neck cancer. Acta Oncol.

